# Anti-AQP4–IgG-positive Leigh syndrome: A case report and review of the literature

**DOI:** 10.3389/fped.2023.1046731

**Published:** 2023-02-06

**Authors:** Jun Chen, Jianjun Wang, Jing Gan, Rong Luo, Zuozhen Yang, Mengmeng Liang, Xiaolu Chen

**Affiliations:** ^1^ Department of Pediatrics, West China Second University Hospital, Sichuan University, Chengdu, China; ^2^ Department of Pediatrics, Key Laboratory of Obstetric & Gynecologic and Pediatric Diseases and Birth Defects of the Ministry of Education, Sichuan University, Chengdu, China; ^3^ Department of Pediatrics, Key Laboratory of Development and Maternal and Child Diseases of Sichuan Province, Sichuan University, Chengdu, China; ^4^ Medical Department, Cipher Gene LLC, Beijing, China

**Keywords:** Leigh syndrome, anti-AQP4–IgG, *MT-ATP6*, m.9176T > C, case report

## Abstract

**Background:**

Leigh syndrome (LS; OMIM: 256000) is a progressive neurodegenerative disease caused by genetic mutations resulting in mitochondrial oxidative phosphorylation defects. The prognosis is poor, with most children dying before the age of 2 years. *MT-ATP6* variants are the most common mitochondrial DNA mutations in LS. *MT-ATP6* variant-induced LS may trigger autoimmunity, and immunotherapy might be effective. Here, we present the first pediatric case of anti-aquaporin 4 (AQP4)–IgG-positive LS caused by an *MT-ATP6* variant.

**Case:**

A 1-year-old boy was hospitalized due to recurrent fever, cough, and developmental regression. Two months previously, he had developed reduced responses to stimulation and psychomotor retardation. After admission, his condition deteriorated and respiratory failure ensued. Magnetic resonance imaging of the brain showed symmetrical small patchy abnormal signals around the third ventricle, pons, and dorsal periaqueductal gray matter in the dorsal medulla. Laboratory tests revealed anti-AQP4–IgG antibodies. Anti-infection, immunoglobulin, and glucocorticoid therapy were administered for symptomatic treatment. Genetic testing revealed a *de novo* homogeneous pathogenic variant of *MT-ATP6* (m.9176T > C, mutation ratio: 99.97%). The patient was diagnosed with anti-AQP4–IgG-positive LS, treated with “cocktail therapy” (vitamins B1, B2, C, and E, l-carnitine, and coenzyme Q10), and discharged after his condition improved. A literature review revealed that LS-induced mitochondrial defects can impact the immune system; hence, immunotherapy and early mitochondrial cocktail therapy may improve outcomes.

**Conclusion:**

Anti-AQP4–IgG-positive LS is very rare. Patients with LS with the m.9176T  > C variant of *MT-ATP6* may be susceptible to autoimmune damage of the central nervous system. Early cocktail therapy combined with immunotherapy may improve their prognosis.

## Introduction

Leigh syndrome (LS), a common mitochondrial disease in infants and children, was first reported by the British neuropathologist Denis A. Leigh in 1951. The incidence of LS is 1 in 40,000 births (1, 2), and the condition invariably carries a poor prognosis. LS typically presents as an early-onset progressive disorder and is caused by defects in mitochondrial oxidative phosphorylation. Its most common clinical characteristics include feeding difficulties, growth retardation, ataxia, psychomotor retardation, dystonia, hypotonia, seizures, and respiratory disorders. The underlying pathology is a subacute necrotizing encephalomyelopathy characterized by bilateral symmetrical necrotic lesions of gray matter nuclei in the basal ganglia, diencephalon, cerebellum, or brainstem. Bilateral lesions in the basal ganglia, thalamus, brainstem (substantia nigra, oculomotor nuclei, periaqueductal gray matter), pontine tegmentum, and inferior olivary nuclei are the most common imaging findings (3). In the majority of cases, the etiology is dysfunction of the mitochondrial respiratory chain caused by either mitochondrial or nuclear gene mutations, which show considerable genetic heterogeneity. The treatment of LS essentially consists of “cocktail therapy” to modulate cell metabolism and correct mitochondrial dysfunction.

Aquaporin-4 (AQP4) is a water-transporting protein expressed in the plasma membrane of astrocytes throughout the central nervous system (CNS). AQP4 is widely involved in diverse functions, such as the regulation of cerebrospinal fluid (CSF) circulation, interstitial fluid resorption, Ca(2+) signaling, potassium buffering, neuroexcitation, and neuroinflammation (4). Therefore, AQP4 is thought to be closely associated with epilepsy, cerebral edema, stroke, multiple sclerosis, neuromyelitis optica spectrum disorder (NMOSD), post-traumatic brain injury, Parkinson disease, and other CNS disorders. AQP4–IgG is generally considered to be the specific autoantibody of NMOSD, which is a rare inflammatory CNS disease that primarily manifests as relapsing episodes of severe optic neuritis and myelitis (5). In this study, we present a very rare case of anti-AQP4–IgG-positive LS with the m.9176T > C variant of the *MT-ATP6* gene*.* The patient exhibited an autoimmune reaction against the CNS, and immunotherapy improved the clinical symptoms.

## Case presentation

**Figure 1:**
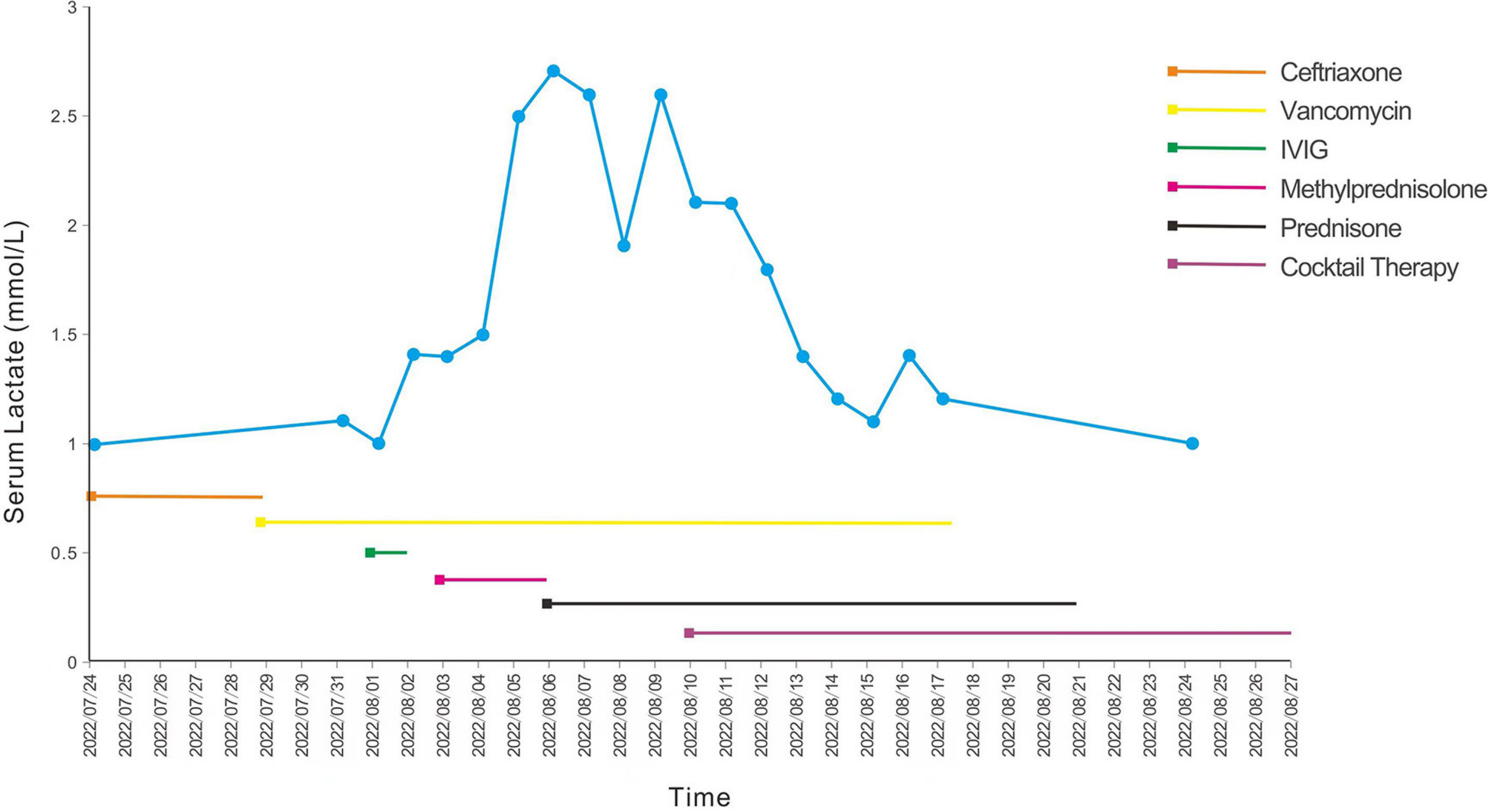
Blood lactate curve and treatment of the patient. The blue line represents the blood lactate levels of the patient after admission, and the straight lines with different colors represent different therapeutic drugs. IVIG, intravenous immunoglobulin.

**Figure 2:**
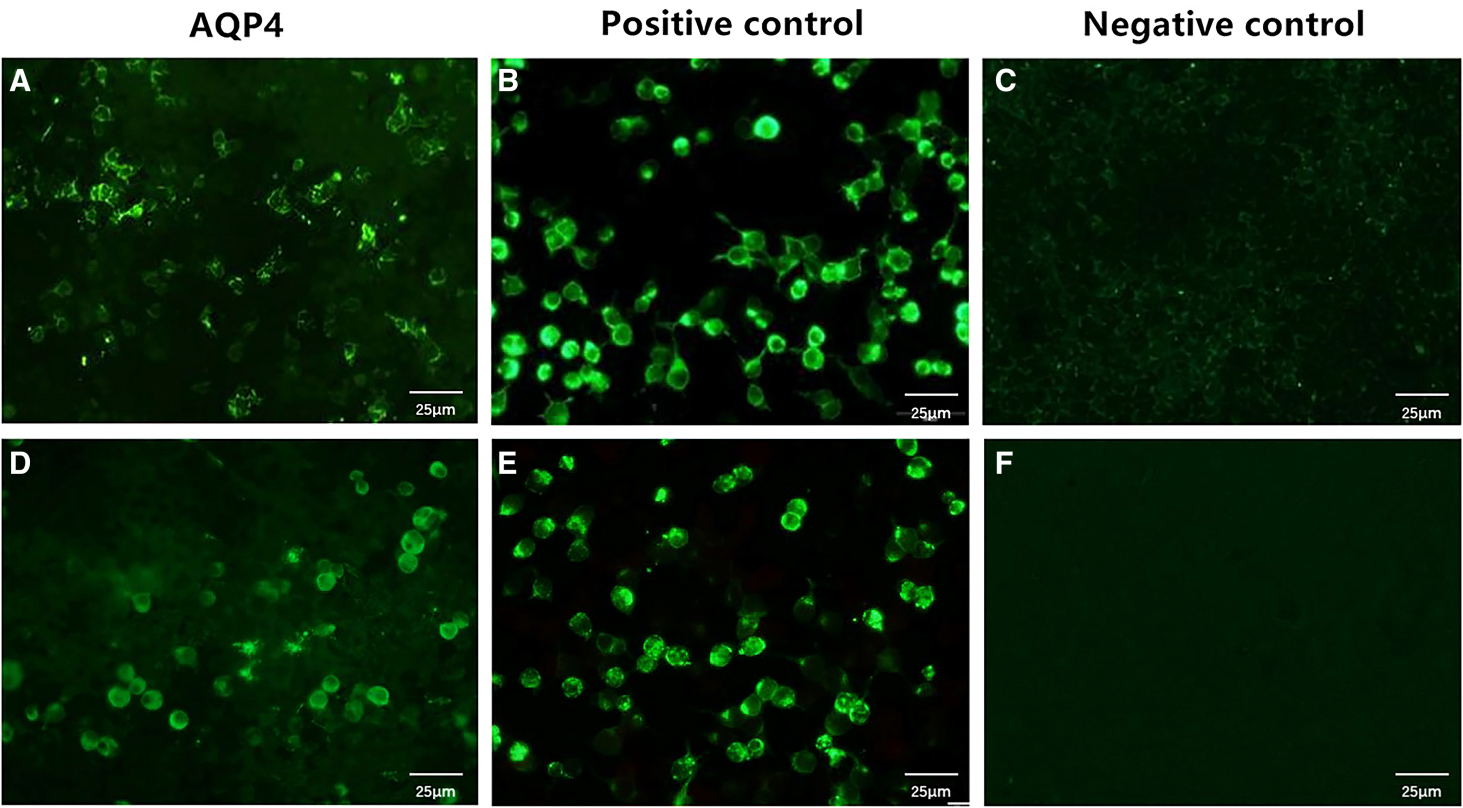
Serum anti-AQP4–IgG levels in the patient. The serum samples of the patient were examined using cell-based assays, which used HEK293 cells transfected with human AQP4. The anti-AQP4 antibody test is positive (**A**), and the antibody titer is 1:10 (initial titer, 1:1). After 3 days, the anti-AQP4 antibody test is still positive, with an antibody titer of 1:10 (**D**). (**B,E**) are positive controls, while (**C,F**) are negative controls. Cells stained green are positive for the anti-AQP4 antibody; unstained cells are negative for this antibody. Scale bar: 25 μm. AQP4, aquaporin-4.

**Figure 3:**
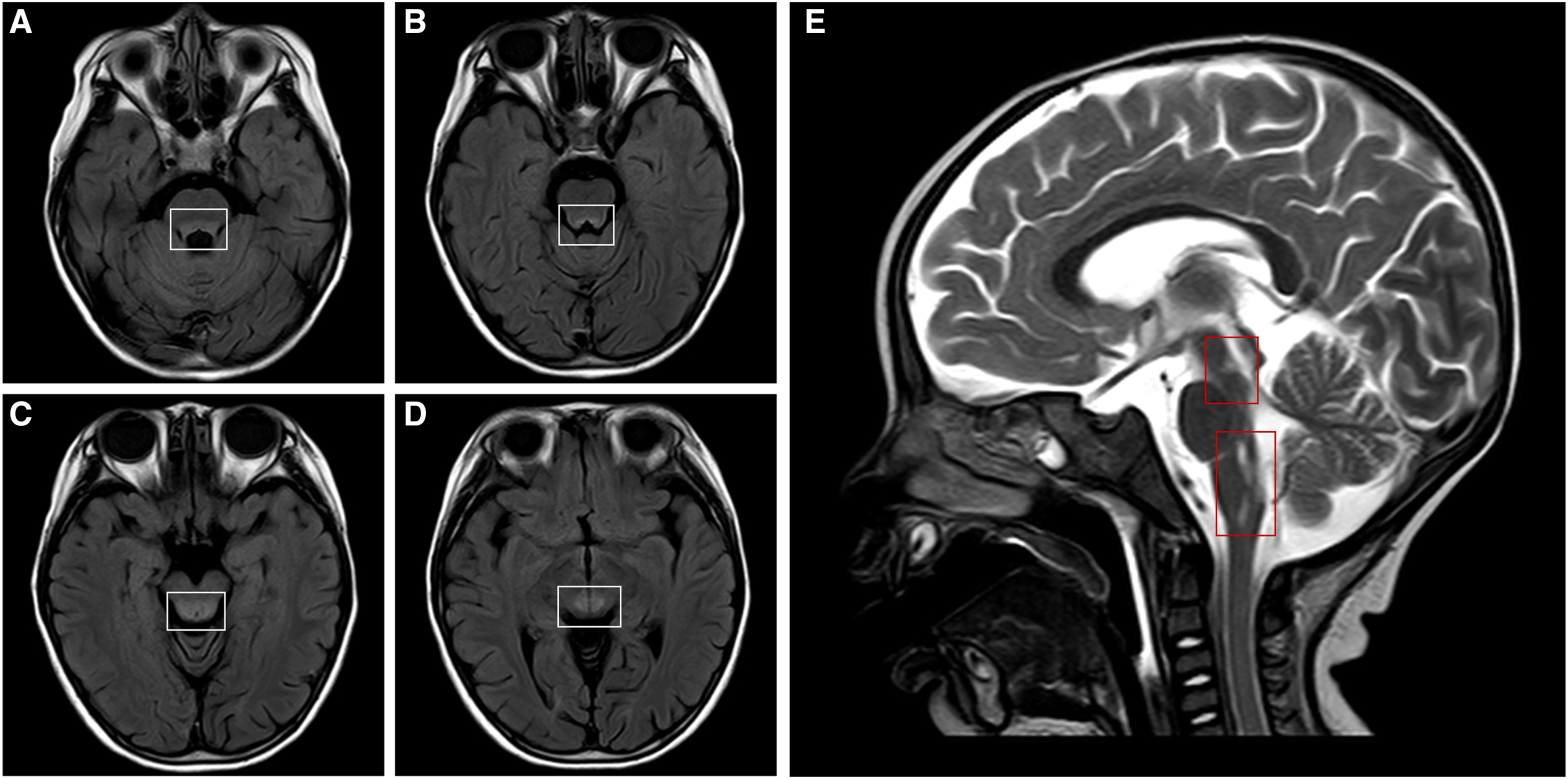
Cranial MRI of the patient. Cranial MRI shows bilateral symmetrical lesions of abnormal signal intensity around the third ventricle, pons, dorsal medulla oblongata, and in the gray matter around the dorsal aqueduct. Axial T2-FLAIR images (**A–D**, white frames) and a sagittal T2-weighted image (**E**, red frames) show the lesions. MRI, magnetic resonance imaging; FLAIR, fluid attenuated inversion recovery.

A 15-month-old boy was admitted to our hospital due to apathetic facial expressions since 2 months, recurrent cough since 1 month, and developmental regression since half a month. Two months previously (at the age of 13 months), the patient appeared to show a lack of proper responses to stimulations, including dull eyes, decreased crying and laughing, and decreased verbal communication with his parents. Then, he developed a monophonic cough accompanied by a slight fever and runny nose. Half a month ago (at the age of 14 months), he exhibited a gradual regression in motor function, and was unable to walk or sit steadily. He was also unable to express himself and could only speak in one- or two-word responses compared with two- to three-word phrases before. The patient's milk intake had decreased from a feed of 150 ml to a feed of 30–40 ml. The patient had no history of other illnesses and no family history of genetic diseases, CNS diseases, autoimmune diseases, developmental delay, or other chronic diseases. A physical examination showed that the patient was awake with apathetic facial expressions. A chest examination revealed rough breath sounds without rales or wheezing. The patient could raise his hands and legs but could not stand or walk steadily due to hypotonia. The Babinski sign was positive on both sides, and the meningeal irritation sign was negative. Laboratory tests performed on admission showed the following: white blood cell count, 19.21 × 10^9^/L (normal range, 4–9 × 10^9^/L); procalcitonin, 0.74 ng/ml (normal range, <0.15 ng/ml); and blood lactate, 4.2 mmol/L (normal range, 0.5–1.7 mmol/L) (Figure 1). Blood cultures were positive for *Staphylococcus hominis* subsp., which was susceptible to vancomycin and levofloxacin. A CSF examination revealed a lactate level of 3.5 mmol/L (normal range, 0.999–2.775 mmol/L); the results of CSF cytology, biochemistry, and culture examinations were all normal. Cell-based assays were performed to assess the serum levels of anti-AQP4–IgG, anti-myelin oligodendrocyte glycoprotein, anti-glial fibrillary acidic protein, and anti-myelin basic protein antibodies: only the serum anti-AQP4–IgG test was positive (Figure 2), with a titer of 1:10 (initial titer, 1:1). Identical oligoclonal bands in the serum and CSF were observed (“mirror pattern,” type 4). Antibodies associated with autoimmune encephalitis were not detected. The auditory brainstem response test showed bilateral prolongation of wave V, and III–V and I–V interwave latencies. The wave amplitudes were decreased. Visual evoked potential testing showed that the P100 wave had a lower amplitude and a latency delay in both eyes. Cranial magnetic resonance imaging (MRI) revealed bilateral symmetrical lesions of abnormal signal intensity around the third ventricle, pons, dorsal medulla oblongata, and in the gray matter around the dorsal aqueduct on T2-weighted images and fluid attenuated inversion recovery (FLAIR) images (Figure 3). Spinal MRI showed no abnormalities in the cervical, thoracic, and lumbar spine. A chest x-ray revealed bilateral pneumonia. An antibiotic treatment regimen of vancomycin was initiated according to the identified bacteria (*Staphylococcus hominis* subsp.) and drug-sensitivity results.

## Results

On day 7 of admission, the condition of the patient worsened; he developed drowsiness, shallow irregular breathing, hypotonia, and fever, which necessitated mechanical ventilation. The patient was transferred to the pediatric intensive care unit due to respiratory failure and unconsciousness (Glasgow coma scale, 10), and was administered invasive mechanical ventilation. Due to the positive anti-AQP4–IgG antibody test, abnormal visual evoked potentials, and abnormal signals on brain MRI, the possibility of NMOSD could not be excluded.

The NMOSD diagnostic criteria issued by the International Panel for Neuromyelitis Optica Diagnosis in 2015 (6) are as follows: (1) patients must have experienced at least one of the six core clinical characteristics of NMOSD, namely, optic neuritis, acute myelitis, area postrema syndrome, acute brainstem syndrome, symptomatic narcolepsy or acute diencephalic clinical syndrome along with NMOSD-typical diencephalic MRI lesions, or symptomatic cerebral syndrome along with NMOSD-typical brain lesions; (2) a positive test for AQP4–IgG (using the best available detection method, such as a cell-based assay); and (3) the exclusion of alternative diagnoses. Our patient had no core clinical characteristics of NMOSD but tested positive for anti-AQP4–IgG. To rule out false-positive results, we reexamined the anti-AQP4–IgG titer 3 days later, and the result was still positive (titer, 1 : 10) (Figure 2).

**Figure 4:**
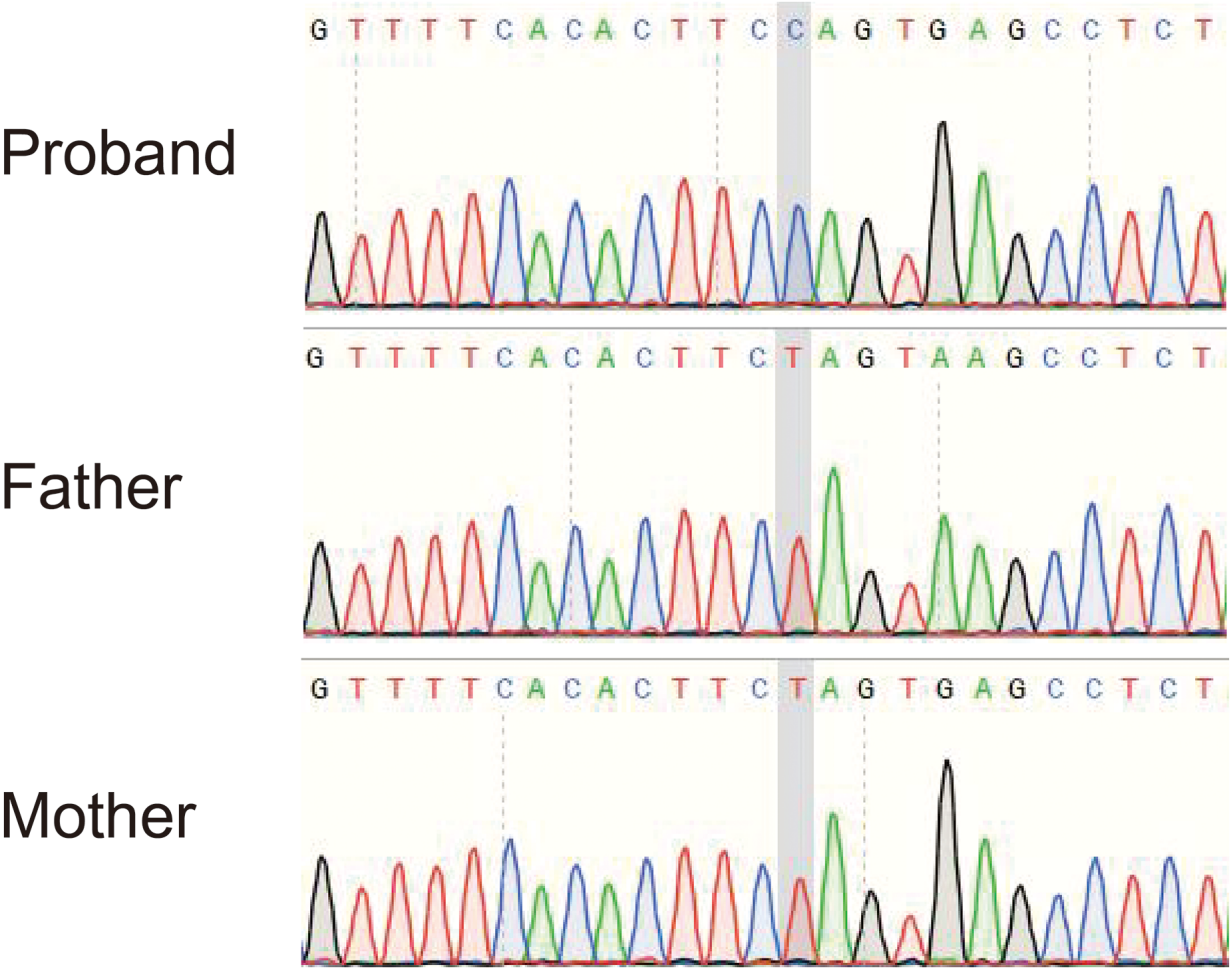
Sequencing result of the *MT-ATP6* gene in the patient. The sequencing analysis revealed a *de novo* homogeneous variant (m.9176T > C) in the *MT-ATP6* gene, which is one of the most common variants in Leigh syndrome.

Although the patient did not meet all the diagnostic criteria for NMOSD, considering that AQP4–IgG is a pathogenic antibody and that autoimmune brain injury could not be completely ruled out, we treated the patient with intravenous immunoglobulin (1 g/kg/day, daily) for 2 days (on days 8 and 9 after admission) and a 3-day course (on days 10–13 after admission) of intravenous methylprednisolone (20 mg/kg/day), followed by oral prednisone (2 mg/kg/day) for 2 weeks (on days 14–28 after admission). After the above treatment, the clinical signs and symptoms of the patient improved, and on day 17 after admission, he was transferred to the pediatric neurology ward for further treatment. Owing to the patient's medical history, clinical manifestations, hyperlactatemia (in serum and CSF), and cranial MRI results, we also considered a diagnosis of the mitochondrial disease LS. Bilateral pneumonia may lead to the deterioration of the primary disease. Therefore, on day 17 after admission, we initiated persistent “cocktail therapy” that included coenzyme Q10 (10 mg/kg/day), l-carnitine (50 mg/kg/day), and multiple vitamins (vitamin B1, vitamin B2, vitamin E, and vitamin C). We also collected peripheral blood samples from the patient and his parents for genetic testing. We detected a *de novo* homogeneous variant in the *MT-ATP6* gene: m.9176T > C (mutation ratio, 99.97%) (Figure 4). This variant is one of the most common variants in this mitochondrial disorder and has been reported as a pathogenic variant in the ClinVar database.

After the diagnosis of LS, we continued to administer the cocktail therapy to the patient. His condition improved, and his serum lactate level decreased (Figure 1); however, he had significant retardation with delayed motor and language development. He also needed to be fed through a gastric tube due to difficulty in swallowing. After a total of 5 weeks in hospital, the patient was finally discharged back home. After being discharged, the patient has continued rehabilitative therapy, and we have suggested a follow-up examination in the outpatient clinic after 3 months (which is yet to occur at the time of writing).

## Discussion and literature review

The onset of LS usually occurs in early infancy or early childhood, and the psychomotor development is usually normal before onset. The main manifestations of LS include psychomotor regression, dystonia, feeding difficulties, ataxia, and brainstem dysfunction (abnormal breathing, ophthalmoplegia, nystagmus, and recurrent vomiting), which are accompanied by increased lactic acid levels in the blood, CSF, and urine (7). The onset of LS is typically induced by a trigger, such as an acute infection or surgery. After the onset of symptoms, the disease advances rapidly, with most patients succumbing to the disease due to respiratory failure within 2 years. The clinical diagnosis of LS requires the following three characteristics (8): (1) neurodegenerative diseases with various clinical manifestations; (2) symmetrical hyperintensities in the basal ganglia and/or brainstem on T2-weighted imaging, and a lactate peak in the affected areas on spectroscopy; and (3) mitochondrial dysfunction caused by a gene mutation.

In this case, the patient developed symptoms at the age of 1 year, and his development was normal before the onset of symptoms. His initial symptoms involved mental status and behavioral changes (such as apathy and a decline in conversation), poor language production, and retardation of motor functions and dystonia, accompanied by elevated lactate levels in the blood and CSF. The imaging findings included bilateral symmetrical hyperintense signal abnormalities in the brainstem and basal ganglia on T2-weighted MRI, which are typical for LS. Genetic tests detected a *de novo* homogeneous pathogenic variant in the *MT-ATP6* gene, namely, m.9176T > C. Thus, the diagnosis of LS was clear in this patient. After the onset of symptoms, the patient's condition rapidly progressed to disorders of consciousness and respiratory failure, necessitating mechanical ventilation.

To date, more than 60 genetic mutations have been reported as causative mutations of LS, of which 70%–80% are nuclear DNA mutations and 10%–20% are mitochondrial DNA (mtDNA) mutations. The mtDNA gene *MT-ATP6* (OMIM: 516,060), which encodes the ATPase6 subunit of the mitochondrial ATP synthase, is located at mtDNA 8527–9207. In this case of LS, the m.9176T > C variant was identified in the *MT-ATP6* gene. To date, 16 cases of LS with the m.9176T > C variant have been reported (Table 1) (9–18). Among these 16 patients, 6 patients were treated with cocktail therapy, of whom 5 patients have survived to date and 1 patient has died (18). In the patient who died, cocktail therapy was begun 2 months after the onset of symptoms, which suggests that early mitochondrial cocktail therapy may improve patients’ outcomes.

**Table 1:** Cases of Leigh syndrome with the T9176C variant of the *MT-ATP6* gene reported in the literature.

Case	Sex	Onset	Clinical features	Cranial imaging	Treatment	Prognosis
Martikainen et al.	Female	1 year	Generalized dystonia of four limbs; chorea	Not done	Not mentioned	Still alive at 13 years
Martikainen et al.	Male	1 year	Generalized dystonia of four limbs	Not done	Not mentioned	Still alive at 14 years
Martikainen et al.	Male	1 year	Generalized dystonia of four limbs; chorea	MRI: Bilaterally increased T2-weighted signals in the putamen, globus pallidus, and dorsal brainstem	Not mentioned	Still alive at 8 years
Dionisi-Vici et al.	Male	9 months	Sudden onset of generalized seizures, hypotonia, and coma during a febrile illness	Not done	Not mentioned	Died a few hours after symptom onset
Dionisi-Vici et al.	Male	9 months	Coma, periodic breathing, seizures, and pyramidal signs following a febrile illness	CT: symmetrical hypodensities in the basal ganglia and brainstem	Not mentioned	Died of cardiorespiratory arrest 3 weeks after admission
Oh et al.	Male	20 years	Bilateral fatigable ptosis	MRI: symmetrical hyperintensities in periaqueductal gray matter on T2- and diffusion-weighted images	Not mentioned	Not mentioned
Ichikawa et al.	Male	10 years	Bilateral ptosis, fluctuating strabismus, ophthalmological symptoms, headache, nausea, and dyspnea	MRI: bilateral high-intensity lesions with restricted diffusion in the dorsal midbrain and medial thalamus and hypothalamus	Cocktail therapy	No progression after 2 years, except for very mild, intermittent right eye exotropia
Campos et al.	Male	3 years	Progressive lethargy, metabolic acidosis, recurrent apnea, responsive only to painful stimuli, diffuse hypotonia, areflexia, and bilateral Babinski sign during a febrile illness	MRI: bilateral T1 and T2 signal increases in the basal ganglia, thalamus, periaqueductal and periventricular regions, and posterior cords of medulla	Not mentioned	Repeated apnea; died 1 month after admission
Liang et al.	Male	12 years	Blepharoptosis, hoarseness, fever, inability to walk and talk; 2 months later, lethargy, dysphagia, limb weakness, and respiratory and circulatory failure occurred	MRI: symmetrical abnormal signals in the basal ganglia, medial thalami, periaqueductal region of the midbrain and pons, and the white matter around both lateral ventricles	Antiviral drugs, dehydration medication, and cocktail therapy	Died of progressive brainstem dysfunction 2 months later
Chuquilin et al.	Female	20 years	Learning disability, hypersomnia, frequent falls, increased headaches with migraine features, intermittent diplopia, bladder incontinence, behavioral changes with apathy, poor hygiene, irritability, and disinhibition	MRI: bilateral hyperintensity in the basal ganglia on T2/FLAIR images	Plasmapheresis, IVIG infusion	Improvements in behavioral symptoms, bladder incontinence, and muscle strength
Jacobs et al.	Female	1 year 9 months	Cerebellar ataxia, speech retardation. After a fever at the age of 3 years, cerebellar ataxia worsened with signs of dystonic posturing of the right arm and leg. At the age of 4 years, the patient developed stomachache, metabolic derangement, and dyspnea	MRI: extensive vaguely demarcated hypodensities in the cerebellar hemispheres, pons, and mesencephalon, and supratentorial hypodense lesions in the caudate nuclei, internal capsule, and basal ganglia	Ventilator	Died of progressive brainstem dysfunction
Jacobs et al.	Male	2 years	Cerebellar ataxia. After viral illnesses at the age of 3 years, the patient developed loss of tone resulting in paraparesis, loss of ambulation, sighing, dyspnea, and dystonic movements	MRI: FLAIR and T2-weighted fast spin echo sequences showed extensive abnormal signals in the cortical and subcortical regions, and cerebellar hemisphere	Cocktail therapy	Cerebellar ataxia, generalized mild dystonic movement disorder, and a discrete pyramidal syndrome at the age of 8 years
Jacobs et al.	Female	2 years	Cerebellar ataxia, speaking in short and poorly articulated sentences	MRI: very discrete abnormalities in the cerebellar hemispheres	Cocktail therapy	Still alive at 6 years
Thyagarajan et al.	Male	4 months	Developmental delay, brisk tendon reflexes, hypotonia, external ophthalmoplegia, oculomotor dyspraxia, unsustained ankle clonus	MRI: bilateral T1 and T2 signal increases in the putamen and periaqueductal regions	Not mentioned	At the age of 6 years, his neurological condition had been static for several years
Thyagarajan et al.	Male	Before 1 year 4 months	Microcephaly, fine and gross motor incoordination. At the age of 8 years, he developed headache and altered mental state after a viral illness	MRI: bilateral hyperintense signals in the striatal region on T1- and T2-weighted images	Not mentioned	Learning disability, poor balance and coordination at the age of 11 years
Wei et al.	Male	27 years	Mental retardation, limited ocular motility of both eyes, ataxia, pyramidal signs, bulbar palsy	MRI: lesions in brainstem, pons, midbrain, periaqueduct, basal ganglia, thalamus, gray matter, putamen, globus pallidus, and caudate nucleus	Cocktail therapy	Still alive
This study	Male	1 year 1 month	Apathetic facial expressions, developmental delay, psychomotor retardation, dystonia, and cardiopulmonary failure	MRI: bilateral symmetrical lesions of abnormal signal intensity around the third ventricle, pons, dorsal medulla oblongata and in the gray matter around the dorsal aqueduct	Cocktail therapy, methylprednisolone, and IVIG	Condition improved and patient discharged with severe psychomotor retardation and difficulty swallowing; rehabilitation after discharge

CT, computed tomography; FLAIR, fluid attenuated inversion recovery; IVIG, intravenous immunoglobulin; MRI, magnetic resonance imaging.

The m.9176T > C variant of the *MT-ATP6* gene results in the replacement of a highly conserved leucine residue (amino acid 270) with a proline residue, which decreases the synthesis of mitochondrial proteins and reduces the activity of oxidative enzymes, causing a decrease in mitochondrial ATP synthesis (19). The reduced ATP production increases the mitochondrial transmembrane potential (with resultant hyperpolarization) and reactive oxygen species production, which in turn activates the necroptosis pathway, resulting in the release of sequestered autoantigens and enhancing the immunogenic properties of neural cells (20). The above findings suggest that secondary cellular necrosis may result in a potential immunoinflammatory reaction in patients with LS with the m.9176T > C variant of the *MT-ATP6* gene.

Our patient also tested positive for serum anti-AQP4–IgG antibodies. In addition, oligoclonal bands were detected in both the serum and CSF of the patient, and his condition improved after intravenous immunoglobulin and glucocorticoid treatment. AQP4–IgG is associated with NMOSD in the majority of cases and has been demonstrated to be directly pathogenic and of high differential diagnostic and prognostic impact (21, 22). The reason for the co-occurrence of LS and anti-AQP4–IgG antibodies in this patient is currently unclear. The AQP4 protein is abundantly expressed in the foot processes of astrocytes that are adjacent to the blood vessels and piamater (around the blood–brain barrier) (23), which is a common site of involvement in LS. A recent study found that mitochondrial dysfunction in the CNS and the peripheral blood may contribute to disease progression and the disability level in patients with NMOSD (24). In patients with LS, mitochondrial dysfunction leads to cell edema and hypoxia, which results in the loss of energy-dependent solute homeostasis and drives water influx down an osmotic gradient into perivascular astrocytes, which ultimately causes swelling (25). Therefore, we speculated that the presence of AQP4 antibodies may be related to the release of autoimmune substances or the exposure of the AQP4 surface antigen secondary to astrocyte edema and necrosis caused by mitochondrial dysfunction. The binding of IgG to AQP4 can lead to the generation of reactive astrocytes and the early recruitment of granulocytes, leading to the amplification of the inflammatory response, reversible internalization of AQP4, direct impairment of water flux independent of antigen downregulation, and downregulation of excitatory amino acid transporter 2, with consequent glutamate excitotoxicity, and complement-dependent and antibody-dependent cellular cytotoxicity that can also damage surrounding cells (5, 26). Increased AQP4–IgG may also worsen mitochondrial dysfunction associated with Na+-K+-ATPase, which could result in increased intracellular crystalloid osmotic pressure and cytotoxic edema.

There is growing evidence that mitochondrial dysfunction can impact every organ system, including the immune system. A recently published study demonstrated that targeting immune cells through high-dose leukocyte-specific inhibitors rescued CNS degeneration in diseases such as LS (27). In addition, plasma exchange synchronized with intravenous gamma globulin therapy was reported to be effective in one patient with LS who also had the m.9176T > C variant of the *MT-ATP6* gene (15). The authors of that case report considered that metabolic damage to neuronal cells may trigger an autoimmune response in mitochondrial diseases. The authors also pointed out that oligoclonal bands have been detected in mitochondrial diseases such as Leber hereditary optic neuropathy, progressive external ophthalmoplegia associated with *POLG* gene mutation mimicking multiple sclerosis, and acute demyelinating encephalomyelitis (28, 29). In one case report, a patient with a confirmed mitochondrial myopathy was found to have muscle T-cell infiltration upon biopsy and showed significant clinical improvement after intravenous immunoglobulin therapy; this case supports the idea of a pathological inflammatory response induced by mitochondrial diseases (30). Mancuso et al. (30) also reported that excellent results were achieved in a patient with mitochondrial myopathy after intravenous immunoglobulin and carnitine treatment, suggesting that mitochondrial diseases might cause a pathological autoimmune inflammatory reaction in the CNS.

Although none of the discussed preclinical therapeutics or clinical immune-targeting therapeutics are approved to treat genetic mitochondrial diseases, corticosteroids can provide significant and at times persistent benefits in genetically and clinically distinct forms of mitochondrial diseases, including MELAS (mitochondrial encephalomyopathy, lactic acidosis, and stroke-like episodes), mitochondrial myopathy, mitochondrial encephalomyopathy, mitochondrial neurogastrointestinal encephalopathy, and mitochondrial leukoencephalopathy (31). Mechanistic target of rapamycin (mTOR) inhibitors, rapamycins, and analogs, such as sirolimus and everolimus, have immunosuppressive actions that are mechanistically distinct from corticosteroids, and these have also been reported to have therapeutic effects in mitochondrial diseases. A 2-year-old patient with LS responded well to mTOR inhibition therapy, with a striking reversal of gross motor function loss and brain lesions, which persisted through 20 months of therapy (32). These findings have dramatically changed our understanding of the pathogenesis of LS, demonstrating that immune involvement is causal in mitochondrial diseases. Further work is needed to elucidate the precise mechanisms between mitochondrial dysfunction, immune dysregulation, and pathology.

## Conclusions

In this study, we have reported a very rare case of anti-AQP4–IgG-positive LS caused by a variant of *MT-ATP6*. Mitochondrial diseases can trigger an autoimmune inflammatory reaction, which influences many organs, especially the CNS. Although previous studies and the present case show that immunotherapy may be effective for the treatment of LS with the m.9176T > C variant, further studies are needed to reveal the intrinsic mechanisms underlying LS with the m.9176T > C variant, which is prone to cause autoimmune injury in the CNS. More cases are needed to verify the efficacy of immunotherapy in this LS subtype.

## Data Availability

The original contributions presented in the study are included in the article/Supplementary Material, further inquiries can be directed to the corresponding author.
